# Sixteen and Sixty (Poetry)

**Published:** 1881-05

**Authors:** 


### SIXTEEN AND SIXTY.

Oh. grandma sits in her oaken chair,
And in flies Bessie with tangled hair.
"I'm going to be married, oh, grandmamma,
I'm going to be married! Ha, ha! Ha, ha!"

Oh, grandma smooths out her apron string;
"Do you know, my dear, 'tis a solemn thing?"
"'Tis solemner not to, grandmamma.
I'm going to be married! Ha, ha! Ha, ha!"

Then grandma looks through her sixty years,
And sums up a woman's hopes and fears:
Six of 'em living and two of 'em dead;
Grandpa helpless and tied to his bed.

Nowhere to live when the house burned down,
Years of fighting with old Mother Brown;
Stockings to darn and bread to bake,
Dishes to wash and dresses to make.

But then the music of pattering feet,
Grandpa's kisses so fond and sweet,
Song and prattle the livelong day,
Joy and kisses and love alway.

Oh, grandma smooths out her apron string,
And gazes down at her wedding ring,
And still she smiles as she drops a tear:
"'Tis solemner not to. Yes, my dear."

We do not think there is the slightest cause for apprehension from the supposed unusual astronomical features of the year 1881, nor do we think there is anything in the Bible to point to the present time as the end of the world. The Bible says nothing whatever to satisfy morbid and unhealthy curiosity on that subject. Allay all your fears and go on with your work, serving God and your fellow man as faithfully, cheerfully and conscientiously as you know how, and you will be ready for the end of the world or any other catastrophe that may come in God's providence whenever the time appointed by God comes round. This in answer to two or three correspondents whose anxieties on this subject probably reflect those of a considerable class of readers. —*Christian Union.*

### PIMPLES.

Pimples are occasioned by the torpid state of the skin, or, in other words, by the inability of the skin to perform its proper functions. The cause of these pimples is, therefore, neither more nor less than an obstruction of the pores of the skin ; the perspiration being allowed to accumulate, the mouths of the pores become clogged, irritation ensues and a pimple arises. The only way to be rid of them is to allow the skin to do its own work by preserving it in a healthy condition and keeping the system in good order. The following ointment is recommended : Take an ounce of barley meal (the finer the better), one ounce of powdered bitter almonds and a sufficient quantity of honey to make them into a smooth paste. If you are taking medicine for the blood adopt our advice and place yourself in the hands of a competent physician.

### HARD USAGE.

As a member of the French diplomatic body was recently traveling to Tehern to join his legation there, he was seized by a band of brigands, who put his uniform on their chief, and turned the fillings of his dressing bag (he was a great Parisian dandy) into useful and ornamental articles of various kinds, according as their fancy suggested. The only thing they could make nothing of was the pomade Hongroise. The unfortunate Frenchman, on being asked what it was, could only reply in Persian, "Butter." The brigands at once tasted the compound, and, concluding a practical joke had been intended, they forced the prisoner at the sword's point to eat the whole of the six pots he had brought with him, and, having completely rifled his baggage and pockets, left him to complete his journey more dead than alive.

THE *Commercial Bulletin* publishes a table showing that there were last year in the United States and Canada 250 fires which involved a loss of $50,000,000 and upward, and ten fires where the loss exceeded $500,000. The aggregate losses by these 250 fires amounted to $35,000,000, or about one-third of the total fire loss of the year. There certainly ought to be some means of preventing this fearful destruction and waste of property. For much of it, no doubt, popular carelessness is responsible ; but defective building methods have a great deal to do with the ravages of fires in all large towns and cities, and these could be cured if the municipal authority was uniformly and vigorously asserted.

